# Unhealthy Snack Food and Beverage Consumption Is Associated with Lower Dietary Adequacy and Length-for-Age *z*-Scores among 12–23-Month-Olds in Kathmandu Valley, Nepal

**DOI:** 10.1093/jn/nxz140

**Published:** 2019-07-16

**Authors:** Alissa M Pries, Andrea M Rehman, Suzanne Filteau, Nisha Sharma, Atul Upadhyay, Elaine L Ferguson

**Affiliations:** 1 Helen Keller International, Washington, DC, USA; 2 Department of Population Health, Faculty of Epidemiology and Population Health, London School of Hygiene and Tropical Medicine, London, United Kingdom; 3 Helen Keller International, Kathmandu, Nepal

**Keywords:** undernutrition, Nepal, unhealthy diet, complementary feeding, snacks

## Abstract

**Background:**

Consumption of unhealthy snack foods and beverages (USFBs) in low- and middle-income countries (LMICs) is rising, with global awareness increasing about risks of overnutrition. However, little is known about the relation between USFB consumption and young children's diet/nutritional outcomes in contexts where nutrient density of complementary foods is often low.

**Objectives:**

This study assessed the association of high USFB consumption, compared with low consumption, with nutrient intakes, dietary adequacy, iron status, and growth in young children in Kathmandu Valley, Nepal.

**Methods:**

A cross-sectional survey was conducted in a representative sample of 745 primary caregivers of children aged 12–23 mo. Food consumption was measured through quantitative 24-h recalls, and child anthropometric measurements and capillary blood samples were collected. Using adjusted linear/logistic regression models, nutrient intakes, dietary adequacy, length-for-age and weight-for-length *z*-scores (LAZ and WLZ, respectively), and iron status were compared between lowest and highest tertiles of consumption based on the contribution of USFBs to total energy intakes (TEIs). Mediation of the relation between USFB consumption and LAZ via lowered dietary adequacy was explored using structural equations modeling.

**Results:**

On average, USFBs contributed 46.9% of TEI among the highest tertile of consumers, compared with 5.2% of TEI among the lowest. Compared with low-USFB consumers, high-USFB consumers had lower nutrient intakes and a greater proportion were at risk of inadequate intakes for 8 nutrients. Mean LAZ was nearly 0.3 SD lower among high-USFB consumers than low consumers (*P* = 0.003), with this relationship partially mediated through dietary adequacy. No associations were found with stunting prevalence or iron status. Prevalence of overweight/obesity was low.

**Conclusions:**

In this LMIC context, high USFB consumption among young children was associated with inadequate micronutrient intakes, which can contribute to poor growth outcomes. Addressing increased availability of USFBs in LMIC food systems should be a priority for policies and programs aiming to safeguard child nutrition.

## Introduction

Recent decades have seen marked growth in global availability of energy-dense, nutrient-poor snack foods and beverages, with consumption rising among adults and children in low- and middle-income countries (LMICs) (1, 2). With escalating rates of noncommunicable diseases (NCDs) and childhood obesity, the role of these foods in overall diets and their deleterious impact on nutrition and health has become a substantial global health concern (3). Whereas the risk of overnutrition among children in high-income settings has been explored (4), there has been less research on how overconsumption of energy-dense/nutrient-poor foods and beverages could potentially lead to micronutrient dilution during a critical period of growth and development. In LMICs where the nutrient density of complementary feeding diets is often low (5), displacement of nutrient-rich foods by unhealthy snack foods and beverages (USFBs) is particularly concerning. However, there is limited research on the correlation between unhealthy diets and nutritional outcomes during the complementary feeding period in these contexts (6).

High consumption of commercially produced snack foods and beverages among infants and children aged <2 y has been noted previously in urban Nepal, with the prevalence of commercial snack food and beverage consumption higher than consumption of micronutrient-rich foods such as eggs and fruits (2). In this context, where nearly one-third of children aged 6–59 mo are stunted, 22% of 6–23-month-olds have iron deficiency anemia (IDA), and only 1 in 2 children achieve the minimum recommendation for dietary diversity during the complementary feeding period (7, 8), such high consumption of USFBs could increase risks of inadequate dietary intakes and the associated negative functional outcomes because of diet displacement. There is a need to understand the role USFBs play in dietary adequacy and nutritional status in this context. The objective of this study, therefore, was to assess associations between high, compared with low, USFB consumption and nutritional outcomes among children aged 12–23 mo in Kathmandu Valley, Nepal, including: nutrient intakes, dietary adequacy, iron status, and anthropometry.

## Methods

### Study design and sampling

A cross-sectional survey was conducted among primary caregivers and their 12–23-month-old children living in Kathmandu Valley, Nepal, from February to April 2017. Participants were selected using 2-stage cluster sampling. In the first stage, 78 clusters were randomly allocated across 1136 municipality wards of Kathmandu Valley based on probability proportional to population size, with larger wards having a higher probability of having more than 1 cluster assigned. In the second stage, a random global positioning system point was identified within the ward for each cluster and 12–13 caregivers were recruited from this starting point 2–3 d prior to data collection using standardized procedures (9), with the assumption that ≤3 children/caregivers would be unavailable on the day of interview due to illness, family emergency, or change of mind. Children/caregivers who did not permanently reside in Kathmandu Valley, and children who were severely ill or had a congenital/physical malformation that inhibited feeding, were excluded. If >1 eligible child lived within a household or if eligible children were from a multiple birth, 1 was randomly selected. Recruited caregivers were contacted on the day of interview to assess availability, and 9–10 available caregivers were interviewed. If >10 of the recruited caregivers in a cluster were available, 10 would be randomly selected for interview. Ethical approval for this study was obtained from the Nepal Health Research Council and the London School of Hygiene and Tropical Medicine. Written informed consent was obtained from all caregivers.

### Study procedures

We collected dietary, anthropometric, and biochemical data for each child, and administered a questionnaire through structured interview with primary caregivers. Interviews and dietary assessments were conducted first within caregivers’ homes to ensure a comfortable environment and also to aid portion size estimation by using household utensils used for cooking/child feeding. Caregivers and children were then brought to a central location within the ward where anthropometric measurements and blood sampling were conducted. All tools were pretested and methods pilot-tested prior to data collection to ensure comprehension and comfort among participants, and interviews were conducted in Nepali.

Questionnaire development was based on prior surveys conducted in Nepal, and included adapted questions from the Nepal Demographic and Health Survey, Nepal Living Standards Survey, and prior surveys conducted in Kathmandu Valley among caregivers of young children (10). Data were collected on demographic and socioeconomic characteristics pertaining to the caregiver and child, as well as factors related to child health and nutrition. Interviewers administered the questionnaires and collected these data electronically on tablets using the open-source online platform ONA (Ona Systems) and Open Data Kit application. Completed questionnaires were submitted to the ONA platform daily and the database downloaded and stored securely. Programmed skip patterns and constraints limited the potential of enumerator error, but data checks were run weekly to ensure data quality.

For the dietary assessment, trained interviewers administered a single, interactive, multiple-pass 24-h recall (24HR) to gather information on the quantities of foods/beverages consumed by the child over the previous day (11). The first pass involved caregivers listing all the foods and drinks consumed by the child in the previous day and the time when they were consumed. During the second pass, the caregiver was asked to provide further details of these foods/drinks, including ingredients in dishes and cooking preparation. In the third pass, caregivers were asked to estimate the portion size of foods and drinks consumed by the child by weighing food/beverage models. Nine different models were used: water, play dough, boiled rice, beaten rice, raw rice, puffed rice, sugar, wheat flour, and peanuts. For some foods, such as packaged items, caregivers were asked to report the number of pieces/packages consumed. A pictorial size guide was also developed for common vegetables and fruits, whereby caregivers could indicate if the size was “small,” “medium,” or “large.” To determine the weight for each of these sizes, the weights of 6 pieces per size per fruit/vegetable were averaged. Additionally, circle models of 3 common sizes of rotis were used for caregivers to estimate the amount fed to their child. In the fourth pass, the interviewer summarized the first pass and verified if the child had consumed anything else; if so, these items were included. To minimize memory error, during recruitment 2–3 d prior to interview, caregivers were given a pictorial food chart and instructed to use it on the specified day prior to data collection. Specifically, caregivers were asked to tick all foods/beverages consumed by their child at the time of consumption and to hand over the pictorial food chart to any other caregivers who watched the child. On the day of interview, interviewers collected these food charts prior to interview and at end of the first pass of the 24HR they crosschecked the foods/beverages recalled by the caregivers with the information ticked off in the food chart and verified any omissions or additions. Dietary data from 24HR were collected on paper forms that were thoroughly reviewed by a supervisor after each interview; when necessary, further clarification from caregivers would be obtained during their visit to the central location for anthropometric measurements/blood sample collection. Data from the paper dietary forms were then entered into an Excel database (Microsoft Corp).

Across the total sample, recalls were conducted on all days of the week to account for day-of-the-week effect at the group level, and a nonconsecutive repeat 24HR was conducted 2–3 d later among 1 randomly sampled child per cluster (*n* = 78; 10.5% of the sample) to account for intraindividual variation. Household utensils and standardized food models were used to estimate portion sizes, which were weighed (±1 g precision) using digital scales (Model KD-810; Tanita Health Equipment). Individual recipes were collected for mixed dishes consumed. Conversion factors were developed to convert quantities measured using food models to actual gram weights consumed. Where caregivers had not been present at the time of a feeding, average recipes calculated from the rest of the sample were used to estimate the grams of ingredients consumed. Energy and nutrient intakes from food/beverages consumed by a child were calculated using a food composition table compiled for this study, which followed guidelines from the FAO International Network of Food Data Systems (INFOODS) guidelines (12) and included a total of 530 foods and beverages. A list of foods that were anticipated to be consumed by children aged 12–23 mo in urban Nepal was initially developed based on review of food composition tables from prior dietary surveys (13) and consultation with local experts. Energy and nutrient values per 100 g of each food/beverage in this list were then compiled from a combination of published food composition tables, including: the Food Composition Table for Bangladesh (14); the US Department of Agriculture National Nutrient Database for Standard Reference (15); the 2015 McCance and Widdowson Composition of Foods Integrated Databases (16); the Association of Southeast Asian Nations Food Composition Database (17); and the Food Composition Database for Nepal (18). Missing values were imputed from similar foods/beverages, with adjustment for differences in moisture content. To account for nutrient losses due to cooking, retention factors were applied as appropriate (14, 15). For commercial products consumed by the children, caregivers were asked to recall brands and flavors, and a market survey was conducted after data collection to capture nutrient content information from these product labels. In addition, energy and nutrient (Ca, Fe, Zn, Na, total fat, sugar, carbohydrate, and protein) content of the 15 most commonly consumed food/beverage products were analyzed by Mahidol University (Bangkok, Thailand) to ensure accuracy during analysis of children's nutrient intakes. These products included: 3 infant cereals, 4 biscuits, 2 chocolates, 2 instant noodles, 2 savory snacks, and 2 sugar-sweetened beverages (SSBs). Multiple packages of each product were purchased in all 3 districts of Kathmandu Valley and across a range of points-of-sale, including large supermarkets, convenience stores, and local corner shops, to ensure wide representation. Product packages were shipped to Mahidol University, where the multiple packages of 1 product were combined for laboratory analysis of energy and nutrient content and duplicate measurements conducted for quality assurance.

Two trained nurses measured the length and weight of each child using standardized procedures (19) with calibrated length boards (ShorrBoards; Weigh and Measure, LLC) and SECA digital scales (model 878 U; ±0·1 kg precision). Two serial measurements of length and weight were taken, and the mean used in analysis. If the 2 measures of length differed by more than 0.5 cm or if weight measures differed by more than 0.5 kg, the measurements were performed again. Children's hemoglobin was measured on site by 2 trained phlebotomists using a HemoCue (model 201+), a portable device that assesses blood hemoglobin concentrations. From each child 150 μL of capillary whole blood was also collected. Pediatric lancets were used for finger pricks and capillary blood collected directly into serum tubes (no. 20.1308; Sarstedt AG & Co. KG). Blood samples were kept appropriately cold and brought to a laboratory in Kathmandu district within 2 h of collection for serum separation and storage. Serum samples were analyzed at the VitMin Laboratory (Willstaett, Germany) using the sandwich ELISA method (20) to assess concentrations of serum ferritin, soluble transferrin receptor (TfR), and 2 acute-phase proteins—C-reactive protein (CRP) and α-1 acid glycoprotein (AGP).

### Exposure and outcomes

Exposure for this study was the highest tertile of USFB consumption compared with the lowest tertile of consumption, with tertiles (low/moderate/high) based on the contribution of these foods to each child's percentage of total energy intake (%TEI) from non–breast-milk foods. Snack foods and beverages were defined based on food type instead of time of consumption or portion size. To create a category of USFB, food types were first categorized as snack foods/beverages and these foods were then nutrient profiled to identify those that were unhealthy. Categorized snack foods and beverages included foods/beverages identified as snacks for young children in prior research (2) and additional Nepal-specific snack foods identified through the formative research (21). This overall category of snack foods/beverages included both homemade and commercially produced items. Within this overall category of snack foods or beverages, foods were then subcategorized as “unhealthy” or “healthy” using a nutrient profiling model from the United Kingdom's Food Standards Agency (UK FSA) (22), which was developed to guide regulation on marketing of unhealthy foods to children. The UK FSA model evaluates the presence and degree of “negative” nutrients (energy, total sugar, saturated fat, and sodium per 100 g) and “positive” nutrients (fiber and protein per 100 g, and percentage fruit/vegetable/nut) to categorize foods as unhealthy or healthy. All foods/beverages were nutrient profiled regardless of source, including those that were commercially produced and homemade.

The primary dietary outcomes compared were: daily intakes of energy and nutrients from non–breast-milk foods; the percentage of children at risk of inadequate intakes of nutrients from both complementary foods and breast-milk; and the mean probability of adequacy (MPA). The prevalence of risk of inadequacy for each USFB consumption tertile was generated for protein and 11 micronutrients (Ca, Fe, Zn, vitamins A, C, B-6, B-12, thiamin, riboflavin, niacin, and folate) using PC Software for Intake Distribution Estimation (PC-SIDE) (23). Estimated nutrient contributions from breast-milk for breastfed children were included in this analysis (24); an estimate of breast-milk intake was calculated by subtracting the median energy intake from non–breast-milk foods from the total energy required for a child equal to the average weight of a breastfed child in our sample. Based on the dietary patterns of children in this study, a low bioavailability of iron and zinc was assumed. The MPA across the 11 micronutrients was also calculated for each child. Best linear unbiased predictor of usual intakes was generated by PC-SIDE for each micronutrient and used to calculate probability of adequacy (PA) for each child using the Institute of Medicine (IOM) probability approach (25). IOM Estimated Average Requirements (EARs) were used for calcium, and vitamins A, C, B-6, B-12, thiamin, riboflavin, niacin, and folate (26), and the International Zinc Nutrition Consultative Group EAR was used for zinc (27). Because iron requirements for young children are not normally distributed, PA was calculated based on probabilities of adequacy for intake intervals (28) and assumed 5% bioavailability.

The primary anthropometric outcomes were *z*-scores for length-for-age (LAZ) and weight-for-length (WLZ), which were calculated using WHO growth standards (29). Stunting was defined as LAZ less than −2 SD and wasting as WLZ less than −2 SD, whereas overweight/obese was defined as WLZ >2 SD. Although a range of micronutrient deficiencies exist among children in Nepal, given a national focus on combating anemia and the substantial prevalence of IDA among 6–23-month-olds, iron status was prioritized for investigation in this study. The primary biochemical outcomes were hemoglobin, TfR, and serum ferritin. Elevation was measured at each municipality ward and used to adjust hemoglobin (30), and concentrations of 2 acute-phase proteins, CRP and AGP, were also measured and serum ferritin was adjusted accordingly for the presence of infection (31). A hemoglobin concentration <11.0 g/dL was categorized as anemia (32). IDA was defined as the presence of anemia, alongside either a low serum ferritin concentration (<12 µg/L) or an elevated TfR concentration (>8.3 mg/L) (32).

### Statistical analysis

Sample size for this study allowed detection of a 0.3 SD difference in nutrient intakes between low and high tertiles of USFB consumption (1-β = 0.9; α = 0.05), as well as a 0.5 difference in LAZ and WLZ (1-β = 0.8; α = 0.05). Data were cleaned and analyzed using STATA-15 (Stata Corp). Data were summarized as proportions or means ± SD for normally distributed data, and medians with IQR for nonnormally distributed data.

Comparisons of nutrient intakes between USFB tertiles were made using cluster-adjusted ANOVA models, and Bonferroni post hoc tests to identify specific differences between low- and high-USFB consumption tertiles. Intergroup comparisons (low- and high-USFB consumers) of the percentage at risk of inadequate nutrient intakes were made using a Pearson chi-square test. Nonnormally distributed outcome data were log transformed prior to analysis. The relations between consumption of USFBs and outcomes related to iron and anthropometric status were explored using linear regression for continuous outcomes and logistic regression for binary outcomes, using random effects to account for cluster sampling. Adjusted models included covariates that are known to influence child nutrition (24); these included caregiver educational attainment, caregiver caste/ethnicity, household wealth status, breastfeeding status, household food security, vitamin A supplementation or deworming in last 6 mo, full immunization status, and child morbidity in last 2 wk. A household wealth index was developed based on principal components analysis, and wealth quintiles were then created (33). The Household Food Insecurity Access Scale was used to categorize children's households as “food secure” or “food insecure (mild/moderate/severe)” (34). Caste/ethnicity was categorized into 4 groups: upper caste/ethnicity (e.g., Brahmin/Chhetri), advantaged janajati (e.g., Newar/Gurung), disadvantaged janajati (e.g., Tamang/Limbu), and other socially disadvantaged groups (e.g., Dalit/religious minorities). Children's age, sex, and birthweight were also included a priori as biological factors assumed to affect nutritional status. Birthweight and immunization status were taken from national health cards (when available) or caregivers’ recall. Variance inflation factors were used to explore colinearity of covariates in the adjusted models; no values exceeded 1.25. The pathway of the relation between USFB consumption and LAZ via dietary adequacy (MPA) was explored with a mediation model using adjusted linear structural equation models. A standardized root mean squared residual was used to evaluate fit of the mediation model, with a value <0.05 indicating adequate fit (35).

## Results

**FIGURE 1:**
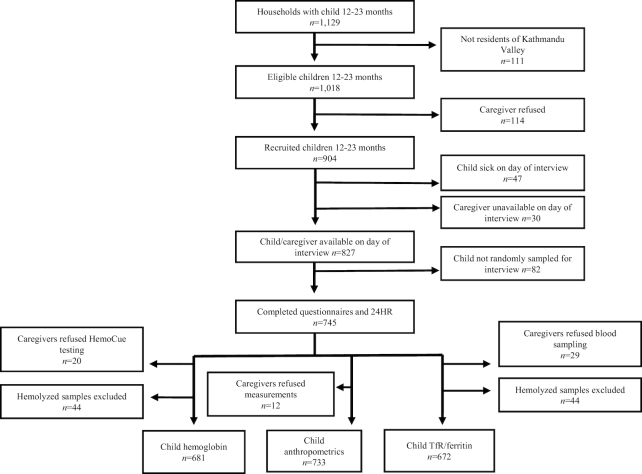
Participant recruitment, exclusion, and inclusion. TfR, soluble transferrin receptor; 24HR, multiple-pass 24-h recall.

Of 1018 eligible caregivers of children aged 12–23 mo identified, 114 (11.2%) declined participation and 77 (7.6%) were lost to follow-up on the day of interview; a final sample of 745 caregivers were interviewed (**Figure 1**). Of these, 733, 725, and 716 caregivers agreed to allow the collection of anthropometric data, hemoglobin, or 150-μL blood samples, respectively. Blood samples from 44 children were excluded from micronutrient status analysis because of hemolysis during processing. There were no statistical differences in demographic characteristics between the children with or without missing biochemical or anthropometric data.

The majority of families were Hindu and from upper caste or advantaged ethnic groups (**Table 1**). Approaching 70% of primary caregivers had at least a secondary level of education, and just over 15% engaged in paid work outside the home. The most common primary caregivers were mothers (90.3%, *n* = 673) and grandmothers (7.1%, *n* = 53). The majority of households were food secure (86.4%, *n* = 644). Nearly 10% of children were low birthweight, and two-thirds had been ill with fever, diarrhea, or cough within 2 wk of the survey. The majority of children were immunized and had received a vitamin A supplement in the past 6 mo, and close to half had been dewormed. Nearly all children were still breastfeeding, with breastfed children receiving a median of 8 breastfeeds (IQR: 6–12) on the day prior to the interview.

**TABLE 1:** Sociodemographic characteristics of Kathmandu Valley primary caregivers and their children aged 12–23 mo (*n =* 745)^1^

	Values
Caregiver characteristics	
Age, y	29.2 ± 8.5
Religion	
Hindu	83.4 (621)
Buddhist	12.3 (92)
Other	4.3 (32)
Ethnic group	
Upper caste	40.3 (300)
Advantaged janajati	26.6 (198)
Disadvantaged janajati	26.2 (195)
Other socially disadvantaged groups	7.0 (52)
Caregiver education	
No formal education	12.8 (95)
Primary	20.3 (151)
Secondary	52.1 (388)
Tertiary	14.9 (111)
Paid work in the last month	30.9 (230)
Works outside the home	16.8 (125)
Child characteristics	
Age, mo	17.6 ± 3.3
Female sex	47.1 (351)
Low birthweight^2^	9.4 (65)
Experienced illness in last 2 wk^3^	66.0 (492)
Preventative health	
Deworming in last 6 mo	48.5 (361)
Vitamin A supplementation in last 6 mo	83.9 (625)
Fully immunized	95.3 (710)

1 Values are mean ± SD or % (*n*).

2 Low birthweight defined as <2.5 kg; birthweight data missing for *n* = 51.

3 Included experiences of fever, cough, or diarrhea.

On average, USFBs contributed 24.5% of TEI from non–breast-milk foods for all children, and contributed on average 5.2%, 21.5%, and 46.9% of TEI for children in the low, moderate, and high tertiles of USFB consumption, respectively. For all children, commercial foods were the main source of energy from USFBs, including biscuits (10.8% TEI), candy/chocolates (3.5% TEI), savory snacks (3.4% TEI), instant noodles (2.2% TEI), and bakery items (2.0% TEI). Commercial SSBs, including fruit drinks, soft drinks, and chocolate beverages, provided 1.2% TEI on average across all children. There was no difference in breastfeeding status (91.2%, 89.5%, and 92.7%; *P* = 0.45) or median number of breastfeeds (9, 8, and 9; *P* = 0.39) across increasing tertiles of USFB consumption. Intakes of 12 nutrients from non–breast-milk foods were significantly higher among low consumers of USFBs compared with high consumers ( **Table 2**). There was little evidence of a difference in energy or fat intakes across USFB consumption tertiles.

**TABLE 2:** Median nutrient intakes from non–breast-milk foods among Kathmandu Valley children aged 12–23 mo, by USFB consumption tertile^1^

	All children (*n* = 745)	Low (*n* = 249)	Moderate (*n* = 248)	High (*n* = 248)	*P* ^2^
Energy, kcal/d	615 (439–855)	666 (459–875)	613 (462–802)	594 (385–833)	0.10
Total fat, g/d	19.9 (12.5–32.3)	20.8 (13.3–33.0)	20.0 (12.6–31.6)	19.3 (11.8–32.2)	0.98
Total protein, g/d	19.0 (12.3–28.5)	23.5 (15.6–33.5)^a^	18.9 (13.0–27.8)^b^	15.7 (10.2–23.2)^c^	<0.001
Calcium, mg/d	245 (111–455)	353 (184–566)^a^	252 (112–455)^b^	161 (67–314)^c^	<0.001
Iron, mg/d	3.5 (2.1–5.6)	4.0 (2.6–6.6)^a^	3.3 (2.3–5.0)^b^	3.0 (1.7–5.0)^b^	<0.001
Zinc, mg/d	2.6 (1.6–4.0)	3.3 (2.2–5.0)^a^	2.5 (1.7–4.0)^b^	1.8 (1.2–3.1)^c^	<0.001
Vitamin C, mg/d	14.0 (5.0–32.2)	17.1 (6.3–34.6)^a^	15.1 (5.6–34.3)^a,b^	9.5 (3.4–25.6)^b^	0.004
Vitamin A (RAE), μg/d	122 (53–227)	170 (88–301)^a^	116 (54–226)^b^	81 (31–171)^c^	<0.001
Thiamin, mg/d	0.3 (0.2–0.6)	0.4 (0.3–0.7)^a^	0.3 (0.2–0.5)^b^	0.3 (0.2–0.5)^b^	0.054
Riboflavin, mg/d	0.6 (0.3–1.1)	0.8 (0.4–1.3)^a^	0.6 (0.3–1.1)^b^	0.5 (0.2–0.8)^b^	<0.001
Niacin, mg/d	4.1 (2.4–6.6)	4.8 (2.9–7.4)^a^	4.2 (2.5–6.7)^b^	3.4 (2.1–5.8)^b^	0.005
Vitamin B-6, mg/d	0.4 (0.2–0.6)	0.4 (0.3–0.7)^a^	0.4 (0.3–0.6)^a^	0.3 (0.2–0.5)^b^	<0.001
Vitamin B-12, μg/d	0.7 (0.3–1.3)	0.9 (0.5–1.5)^a^	0.7 (0.3–1.3)^b^	0.6 (0.2–1.0)^b^	<0.001
Folate, μg/d	57.2 (32.7–93.6)	72.0 (43.3–120.9)^a^	57.4 (34.1–90.6)^b^	47.6 (25.2–71.2)^c^	<0.001

1 Values presented as median (IQR). ANOVA of log-transformed data with cluster adjustment used and Bonferroni post hoc tests conducted to compare between groups; labeled medians in a row without a common letter differ, *P* < 0.05. Low consumption = children in lowest tertile of percentage of total energy intake (%TEI) from unhealthy snack foods and beverages (USFBs) (mean = 5.2% TEI); moderate consumption = children in moderate tertile of %TEI from USFBs (mean = 21.5% TEI); high consumption = children in highest tertile of %TEI from USFBs (mean = 46.9% TEI). RAE, retinol activity equivalents.

2 Overall *P* value of effect of USFB consumption tertiles on nutrient intakes.

**FIGURE 2:**
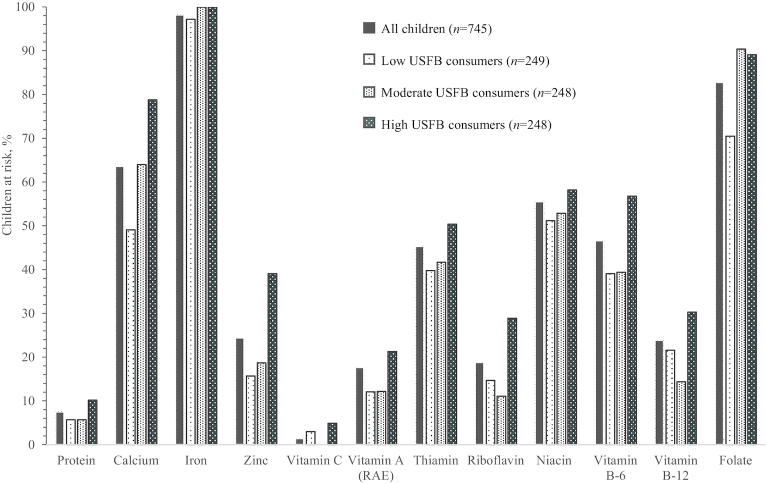
Proportion of Kathmandu Valley children aged 12–23 mo at risk of inadequate nutrient intakes, by tertiles of USFB consumption. Includes estimated nutrient intakes from breast milk. Vitamin C intake for middle USFB consumption tertile could not be normalized. RAE, retinol activity equivalents; USFB, unhealthy snack food and beverage.

Half of all children were at risk of inadequate thiamin, niacin, and vitamin B-6 intakes, two-thirds were at risk of inadequate calcium intakes, and most were at risk of inadequate iron and folate intakes (**Figure 2**). A significantly higher proportion of high-USFB consumers were at risk of inadequate intake for 8 nutrients compared with low-USFB consumers, including: calcium (*P* < 0.001), zinc (*P* < 0.001), vitamin A (*P* = 0.005), thiamin (*P* = 0.02), riboflavin (*P* < 0.001), vitamin B-6 (*P* < 0.001), vitamin B-12 (*P* = 0.03), and folate (*P* < 0.001). Nearly all children were at risk of inadequate iron intake, regardless of USFB consumption tertile. Of the 672 children whose iron status was assessed, over one-third were anemic and nearly one-third had IDA (**Table 3**).

**TABLE 3:** Nutritional status outcomes of Kathmandu Valley 12–23-month-olds, by tertiles of USFB consumption^1^

	All children	Low USFB consumption (5.2% TEI)	Moderate USFB consumption (21.5% TEI)	High USFB consumption (46.9% TEI)
Iron status				
*n*	681	222	227	232
Hb, g/dL	11.2 ± 1.1	11.2 ± 1.1	11.2 ± 1.2	11.1 ± 1.1
Anemia (Hb <11.0 g/dL)	37.7 (257)	35.1 (78)	39.2 (89)	38.8 (90)
* n*	672	216	224	232
Serum ferritin, μg/L	15.6 (8.8–24.6)	14.8 (8.5–24.2)	14.7 (8.7–25.1)	16.8 (9.2–24.2)
Low serum ferritin (<12.0 μg/L)	36.9 (248)	38.0 (82)	38.4 (86)	34.5 (80)
TfR, mg/L	8.6 (7.4–10.7)	8.4 (7.4–11.0)	8.7 (7.5–10.1)	8.5 (7.3–10.8)
High TfR (>8.3 mg/L)	56.1 (377)	53.7 (116)	59.4 (133)	55.2 (128)
IDA	28.7 (193)	27.3 (59)	30.4 (68)	28.5 (66)
Anthropometric status				
* n*	733	246	242	245
LAZ	−0.93 ± 1.09	−0.75 ± 1.15	−0.93 ± 1.04	−1.12 ± 1.06
Stunting (LAZ less than −2)	18.8 (138)	15.9 (39)	18.6 (45)	22.0 (54)
WLZ^2^	−0.42 ± 1.00	−0.44 ± 1.08	−0.43 ± 1.00	−0.41 ± 0.93
Wasting (WLZ less than −2)^2^	5.2 (38)	6.1 (15)	5.4 (13)	4.1 (10)
Overweight/obese (WLZ >2)^2^	0.6 (4)	0.4 (1)	0.8 (2)	0.4 (1)

1 Tertiles of consumption based on percentage of total energy intake (TEI) from unhealthy snack foods and beverages (USFBs). Values presented as mean ± SD; median (IQR); or % (*n*). Hb, hemoglobin; IDA, iron deficiency anemia; LAZ, length-for-age *z*-score; TfR, soluble transferrin receptor; WLZ, weight-for-length *z*-score.

2 One WLZ value excluded as an outlier; *n* = 732 for all children and *n* = 245 for low-USFB consumption group.

One-fifth of children were stunted whereas <6% of children were wasted, and prevalence of overweight/obesity was very low (Table 3). High USFB consumption was negatively associated with LAZ, whereas it was not significantly associated with iron status, WLZ, stunting, or wasting (**Table 4**). In adjusted models, the LAZ of children who consumed a high %TEI from USFBs were 0.3 SD lower than those who had low USFB consumption. In the adjusted model, the overall significance of all 3 USFB consumption tertiles for LAZ was *P* = 0.013, with mean LAZ −0.13 lower in moderate-USFB consumers compared with low-USFB consumers. Table 4 presents results comparing high and low USFB consumption tertiles; model results comparing moderate with low tertiles are presented in **Supplemental Table 1**.

**TABLE 4:** Effect of high versus low USFB consumption on iron and anthropometric status outcomes among Kathmandu Valley 12–23-month-olds^1^

	Unadjusted^2^			Adjusted^2^, ^3^		
	*n*	β/OR (95% CI)	*P*	*n*	β/OR (95% CI)	*P*
Iron status						
Hb, g/dL	681	β = −0.08 (−0.29, 0.13)	0.44	639	β = −0.09 (−0.32, 0.14)	0.44
Serum ferritin, μg/L	672	β = 0.09 (−0.04, 0.22)	0.20	632	β = 0.08 (−0.07, 0.23)	0.28
TfR, mg/L	672	β = −0.02 (−0.07, 0.04)	0.58	632	β = 0.01 (−0.05, 0.06)	0.84
IDA	672	OR = 1.06 (0.70, 1.60)	0.79	632	OR = 1.27 (0.78, 2.07)	0.34
Anthropometric status^4^						
LAZ	733	β = −0.37 (−0.56, −0.18)	<0.001	684	β = −0.29 (−0.49, −0.10)	0.003
WLZ^5^	732	β = 0.03 (−0.15, 0.21)	0.77	683	β = −0.09 (−0.28, 0.10)	0.37
Stunting (LAZ less than −2)	733	OR = 1.51 (0.95, 2.39)	0.08	684	OR = 1.25 (0.70, 2.24)	0.45
Wasting^5^ (WLZ less than −2)	732	OR = 0.65 (0.29, 1.48)	0.31	683	OR = 1.11 (0.40, 3.04)	0.84

1 High consumption: 46.9% of total energy intake (TEI) from unhealthy snack foods and beverages (USFBs); low consumption: 5.2% TEI from USFBs. Hb, hemoglobin; IDA, iron deficiency anemia; LAZ, length-for-age *z*-score; TfR, soluble transferrin receptor; WLZ, weight-for-length *z*-score.

2 Comparisons between high and low snack consumers made using random-effects linear and logistic regression with cluster adjustment.

3 Adjusted for: child age, sex, morbidity, deworming, immunization status, vitamin A supplementation, birthweight, breastfeeding status, caste/ethnicity, caregiver education, household food security, and wealth status.

4 Children with length/weight measurements but without birthweight data missing (*n* = 49) from anthropometric adjusted models.

5 One WLZ value excluded as an outlier.

Results from mediation analysis suggest that dietary adequacy (MPA) partially mediated the relationship between high USFB consumption and lower LAZ. An increase in each USFB consumption tertile was associated with a decrease in MPA of 5% (*P* < 0.001), whereas a 10% increase in MPA was associated with an increase of 0.04 SD in LAZ (*P* = 0.041). The indirect effect of USFB via MPA (β = −0.02; *P* = 0.065) accounted for 13.3% of the total effect (β = −0.15; *P* = 0.005) of USFB consumption on child LAZ. The mediation pathway from USFB consumption to child LAZ via dietary adequacy (defined as MPA) is summarized in **Supplemental Figure 1**.

## Discussion

Few studies have assessed the relationship between USFB consumption and nutritional outcomes in children aged <2 y in a LMIC (6); to our knowledge, this is the first study to comprehensively assess this relationship across a range of nutritional outcomes, including: nutrient intakes, dietary adequacy, iron status, and growth. In this study setting of the Kathmandu Valley, high-USFB consumers were at higher risk of inadequate intakes for 8 micronutrients and had significantly lower mean LAZ than low-USFB consumers. There was no association between USFB consumption and overweight/obesity. Whereas prior research has primarily focused on the relationship between the consumption of unhealthy foods and child overnutrition, our study indicated that an unhealthy dietary pattern in young children can contribute to poor linear growth in contexts where the nutrient density of complementary foods is low and a high proportion of children are at risk of inadequate micronutrient intakes.

Our findings in Kathmandu Valley support the theory that displacement of traditional foods by USFBs could be diluting the micronutrient content of diets during the complementary feeding period. This study builds on prior studies of older children living in both high-income and moderate-to-low–income countries. High intakes of added sugar were negatively associated with micronutrient intakes among 1–3-year-old South African children (36), and high snack consumption was associated with low intakes of protein and vitamin C in rural Indonesian school children (37). In Australian 16–24-month-olds, high consumption of energy-dense and nutrient-poor foods was inversely associated with dietary intakes of micronutrients (38). In LMIC settings, where the micronutrient content of complementary foods is typically low, the displacement of traditional foods by USFBs is particularly concerning for the health and development of young children.

In our study, mean LAZ was significantly lower with increasing tertiles of USFB consumption whereas the prevalence of overweight/obesity was low, suggesting that high USFB consumption contributes to linear growth faltering but not excessive weight gain during the complementary feeding period in children in urban Nepal. These results from Kathmandu Valley challenge the current assumption that overnutrition is the main outcome of concern when USFB consumption is high. In populations where most children are at risk of inadequate nutrient intakes and the prevalence of overweight/obesity is low, micronutrient deficiencies and poor linear growth are instead major concerns. Our findings contrast with a previous study that did not find an association between snack food consumption and LAZ among 6–12-month-olds in periurban South Africa (39) when children who consumed USFBs daily were compared with those who did not. This study, however, did not consider the quantity of snack foods consumed or their contribution to overall dietary adequacy, as indicative of micronutrient displacement. Additional research is needed to build the body of evidence around this relationship.

High USFB consumption was not associated with overweight/obesity or mean WLZ among children in our study. These results agree with a South African study that showed no association between BMI *z*-score and unhealthy food consumption in 12-month-olds (39). However, our findings contrast with a Mexican study that found that 5–24-month-olds who consumed high-fat snacks or sweetened drinks in the previous week were at higher odds of being overweight/obese than other children (40). Ultimately, the different measures used for food consumption in these studies (i.e., weekly or daily consumption without consideration of overall dietary intake) pose a challenge for comparison with our study. Further, the prevalence of overweight/obesity in our study was very low compared with the Mexico study (<1% compared with 19%). Our study also was not powered to detect associations with anthropometric status as categorical outcomes. Despite the low prevalence of overweight/obesity observed in our study, the dietary pattern of these Kathmandu Valley children is cause for concern. In Nepal, the prevalence of adult obesity (7) and diabetes (41) is increasing, which indicates that these unhealthy eating patterns in young urban Nepali children urgently need to be addressed.

Our dietary results suggest that 1–2-year-old children living in the Kathmandu Valley are at high risk of multiple micronutrient deficiencies, which are further exacerbated by high intakes of USFBs. The biochemical analyses conducted for this study confirm that iron deficiency is common. IDA affected nearly one-third of children in our study, which is comparable to the 2016 national rates of IDA among 6–23-month-olds (22%) (8). The lack of association between iron status and consumption of USFBs, however, is not surprising because nearly all the children were at risk of inadequate dietary intakes of iron. Further, our dietary results point to a need to investigate the biochemical status of multiple micronutrients in this population, where >20% of children were at risk of inadequate intakes of thiamin, niacin, folate, and vitamins B-6 and B-12. Investigation into the biochemical zinc status of high- compared with low-USFB consumers is also warranted given the role of this nutrient in child growth and the negative association found between USFB consumption and LAZ.

There are several limitations to this study. First, the cross-sectional design prevents inference of a causal relationship between the USFB consumption observed in children in this study and their nutritional outcomes. However, our results present a plausible biological pathway for this relationship by showing that lower nutrient intakes/higher risk of dietary inadequacy, and ultimately poorer growth outcomes were present in high- compared with low-USFB consumers. Secondly, we did not directly measure breast-milk consumption but instead estimated intakes at the population level. Although we took into account these estimated breast-milk intakes when assessing dietary adequacy and controlled for breastfeeding status in analyses, this source of error could attenuate associations between dietary intakes and other variables. Future research would benefit from more precise quantification of breast-milk consumption. Finally, this study was not powered to detect intergroup differences in the categorical statuses of IDA, stunting, overweight/obesity, or wasting. A positive trend was found in the prevalence of stunting across USFB consumption tertiles, which was consistent with the negative association observed with LAZ; however, it was not significant. Future studies are needed, including cohort studies that can provide greater understanding of causality and studies that are adequately powered for categorical outcomes.

With growing efforts aimed to address the role of unhealthy foods and beverages in the rise of child overnutrition, results from this study call for researchers, program implementers, and policy-makers to expand our understanding of and actions regarding the potential impact of these foods on younger children. Although snacks are an important part of complementary feeding practices, programs should revisit definitions of recommended snacks and consider discouraging unhealthy foods, not only to prevent child obesity but also to safeguard nutritious diets for growth and development. Our results suggest that rapidly changing food environments and subsequent dietary patterns in LMICs (1) might curtail recent achievements in the reduction of childhood stunting in LMICs. There is a need to support caregivers in making healthy food choices (42), and to incorporate messaging about the poor nutritional value of unhealthy commercially packaged snack foods/beverages into nutrition education. Although some of the USFBs were fortified, including several instant noodles and biscuits, their high added sugar/sodium content outweighed the micronutrient density of these products during nutrient profiling and indicates that the overall healthfulness of fortified foods needs to be considered. There is also a crucial need for further research on the role of these foods in young children's diets and the consequent nutritional and functional outcomes. If such unhealthy food products are not only contributing to rising rates of obesity and NCDs but also are associated with poor growth and development, there is an even greater need to enact and enforce marketing regulations to reduce their harmful nutritional impact.

## Supplementary Material

nxz140_Supplemental_Files

## Notes

Funding was provided by the Bill & Melinda Gates Foundation.

Author disclosures: AMP, AMR, SF, NS, AU, and ELF, no conflicts of interest.

Supplemental Table 1 and Supplemental Figure 1 are available from the “Supplementary data” link in the online posting of the article and from the same link in the online table of contents at https://academic.oup.com/jn/.

Abbreviations used: AGP, α-1 acid glycoprotein; CRP, C-reactive protein; EAR, Estimated Average Requirement; IDA, iron deficiency anemia; IOM, Institute of Medicine; LAZ, length-for-age *z*-score; LMIC, low- and middle-income country; MPA, mean probability of adequacy; NCD, noncommunicable disease; PA, probability of adequacy; PC-SIDE, PC Software for Intake Distribution Estimation; SSB, sugar-sweetened beverage; TEI, total energy intake from non–breast-milk foods; TfR, soluble transferrin receptor; UK FSA, United Kingdom Food Standards Agency; USFB, unhealthy snack food and beverage; WLZ, weight-for-length *z*-score; 24HR, multiple-pass 24-h recall; %TEI, percentage of total energy intake from non–breast-milk foods.
