# Supplementary material for: Unhealthy Snack Food and Beverage Consumption Is Associated with Lower Dietary Adequacy and Length-for-Age z-Scores among 12–23-Month-Olds in Kathmandu Valley, Nepal
Source: J Nutr. 2019 Jul 16;149(10):1843–51. doi: 10.1093/jn/nxz140 (PMC6768809; doi:10.1093/jn/nxz140)

Supplementary data

Supplemental figure 1. Mediation analysis of association between USFB consumption and child LAZ via dietary adequacy (MPA) among Kathmandu Valley children 12-23 months of age (*n*=684). Linear structural equations modeling used for analysis, with adjustment for: child age, sex, morbidity, deworming, immunization status, vitamin A supplementation, birthweight, breastfeeding status, caste/ethnicity, caregiver education, household food security, wealth status. SRMR=0.005. LAZ, length-for-age z-score; MPA, mean probability of adequacy; SRMR, standardized root mean squared residual; USFB, unhealthy snack foods and beverages.

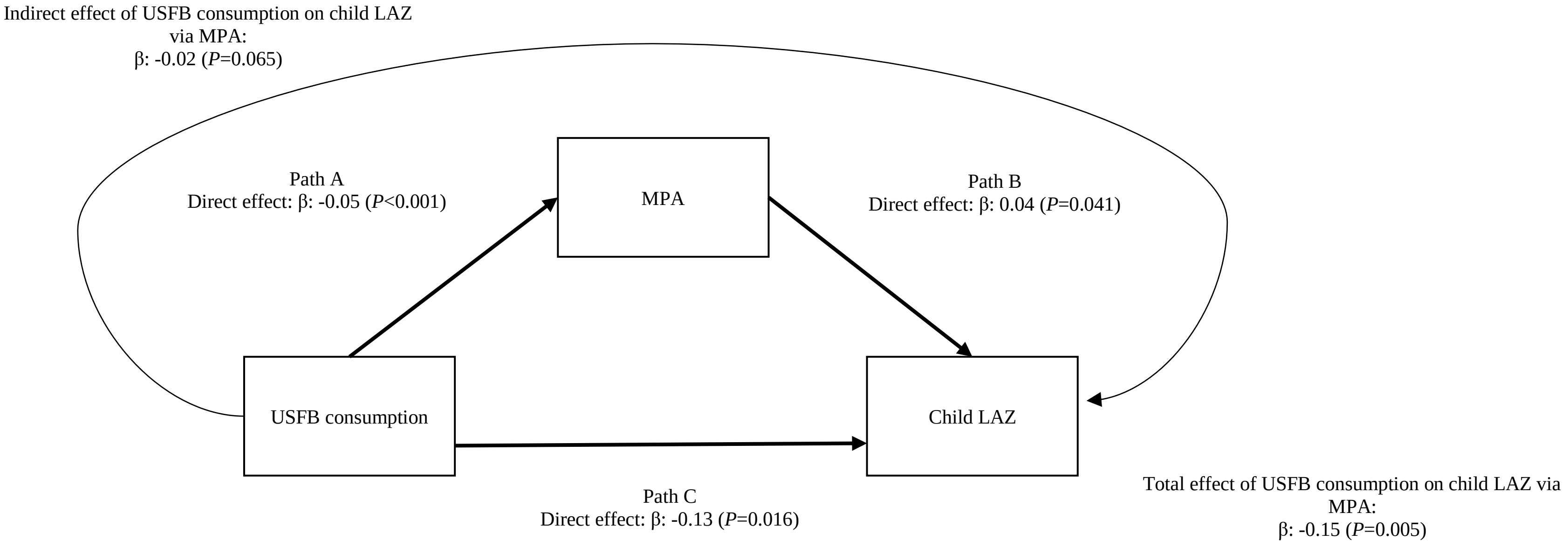

Supplement: nxz140_Supplemental_Files [file nxz140_supplemental_files.zip › Figure S1_JoN May 1 2019.pdf]
