# Supplementary material for: Unhealthy Snack Food and Beverage Consumption Is Associated with Lower Dietary Adequacy and Length-for-Age z-Scores among 12–23-Month-Olds in Kathmandu Valley, Nepal
Source: J Nutr. 2019 Jul 16;149(10):1843–51. doi: 10.1093/jn/nxz140 (PMC6768809; doi:10.1093/jn/nxz140)
Supplement: nxz140_Supplemental_Files [file nxz140_supplemental_files.zip › Table S1_JoN_May 28 2019.pdf]

Supplementary data

Supplemental Table 1. Effect of middle vs. low USFB consumption on iron and anthropometric status outcomes[1]

	Unadjusted[2]			Adjusted[2,3]		
**Iron status**						
	n	**β/OR (95% CI)**	***P***	***n***	**β/OR (95% CI)**	***P***
Haemoglobin (g/dL)	681	β: -0.04 (-0.25 – 0.16)	0.67	639	β: -0.04 (-0.26 – 0.18)	0.71
Serum ferritin (μg/L)	672	β: 0.05 (-0.08 – 0.18)	0.47	632	β: 0.03 (-0.11 – 0.17)	0.66
Transferrin receptor (mg/L)	672	β: -0.004 (-0.06 – 0.05)	0.89	632	β: 0.001 (-0.05 – 0.06)	0.94
Iron-deficiency anaemia	672	OR: 1.16 (0.77 – 1.75)	0.48	632	OR: 1.33 (0.84 – 2.09)	0.22
**Anthropometric status[4]**						
	n	**β/OR (95% CI)**	***P***	***n***	**β/OR (95% CI)**	***P***
Length-for-age z-score (LAZ)	733	β: -0.18 (-0.37 – 0.01)	0.07	684	β: -0.13 (-0.32 – 0.05)	0.15
Weight-for-length z-score (WLZ)[5]	732	β: 0.001 (-0.17 – 0.18)	0.99	683	β: -0.08 (-0.26 – 0.09)	0.36
Stunting (LAZ < -2)	733	OR: 1.22 (0.76 – 1.95)	0.42	684	OR: 0.99 (0.57 – 1.73)	0.98
Wasting[5] (WLZ < -2)	732	OR: 0.87 (0.41 – 1.87)	0.72	683	OR: 1.01 (0.42 – 2.48)	0.98

[1]Middle consumption: 21.5% of total energy intake (TEI) from unhealthy snack foods and beverages (USFB); low consumption: 5.2% TEI from USFB

[2]Comparisons between middle and low snack consumers made using random-effects linear and logistic regression with cluster adjustment

[3]Adjusted for: child age, sex, morbidity, deworming, immunization status, vitamin A supplementation, birthweight, breastfeeding status, caste/ethnicity, caregiver education, household food security and wealth status

[4]Children with length/weight measurements but without birthweight data missing (n=49) from anthropometric adjusted models

[5]One WLZ value excluded as an outlier
